# Lipophilic Cations Rescue the Growth of Yeast under the Conditions of Glycolysis Overflow

**DOI:** 10.3390/biom10091345

**Published:** 2020-09-20

**Authors:** Svyatoslav S. Sokolov, Ekaterina A. Smirnova, Olga V. Markova, Natalya A. Kireeva, Roman S. Kirsanov, Liudmila S. Khailova, Dmitry A. Knorre, Fedor F. Severin

**Affiliations:** 1 Department of Molecular Energetics of Microorganisms, Belozersky Institute of Physico-Chemical Biology, Lomonosov Moscow State University, 1-40 Leninskie Gory, 119991 Moscow, Russia; sviatoslav.sokolov@gmail.com (S.S.S.); smirka@belozersky.msu.ru (E.A.S.); markova@genebee.msu.ru (O.V.M.); kirsanov_roman@mail.ru (R.S.K.); khailova@genebee.msu.ru (L.S.K.); knorre@belozersky.msu.ru (D.A.K.); 2 Department of Soil Biology, Faculty of Soil Science, Lomonosov Moscow State University, 1-12 Leninskie Gory, 119234 Moscow, Russia; nkomarova.95@gmail.com; 3 Institute of Molecular Medicine, Sechenov First Moscow State Medical University, 8/2 Trubetskaya Str., 119991 Moscow, Russia

**Keywords:** yeast, mitochondria, membrane potential, glycolysis, uncoupler

## Abstract

Chemicals inducing a mild decrease in the ATP/ADP ratio are considered as caloric restriction mimetics as well as treatments against obesity. Screening for such chemicals in animal model systems requires a lot of time and labor. Here, we present a system for the rapid screening of non-toxic substances causing such a de-energization of cells. We looked for chemicals allowing the growth of yeast lacking trehalose phosphate synthase on a non-fermentable carbon source in the presence of glucose. Under such conditions, the cells cannot grow because the cellular phosphate is mostly being used to phosphorylate the sugars in upper glycolysis, while the biosynthesis of bisphosphoglycerate is blocked. We reasoned that by decreasing the ATP/ADP ratio, one might prevent the phosphorylation of the sugars and also boost bisphosphoglycerate synthesis by providing the substrate, i.e., inorganic phosphate. We confirmed that a complete inhibition of oxidative phosphorylation alleviates the block. As our system includes a non-fermentable carbon source, only the chemicals that did not cause a complete block of mitochondrial ATP synthesis allowed the initial depletion of glucose followed by respiratory growth. Using this system, we found two novel compounds, dodecylmethyl diphenylamine (FS1) and diethyl (tetradecyl) phenyl ammonium bromide (Kor105), which possess a mild membrane-depolarizing activity.

## 1. Introduction

A mild decrease in the ATP/ADP ratio can be beneficial for cells [1]. Such a decrease could be achieved by various means, i.e., by adding chemicals dissipating mitochondrial membrane potential [1,2], moderating the mitochondrial respiratory machinery [3] or decreasing the rate of glycolysis (reviewed in [4]). Such chemicals are promising drugs for treating obesity; they also can be considered as caloric restriction mimetics.

**Figure 1:**
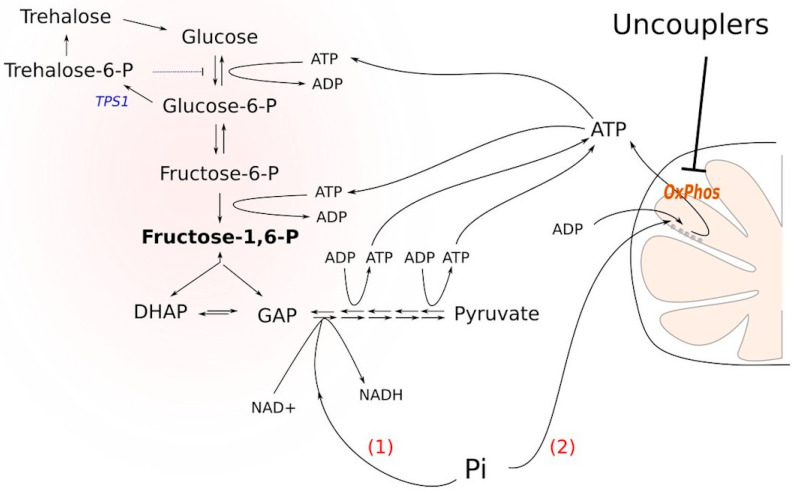
Two routes of inorganic phosphate entry into cell catabolism under glycolysis-overflow conditions induced by *TPS1* deletion. (1) Glyceraldehyde dehydrogenase reaction consumes Pi and induces upstream fructose-1,6-bisphosphate processing. This route is expected to alleviate glycolytic block. (2) ATP synthesis via oxidative phosphorylation (OxPhos) consumes Pi; this route can aggravate glycolytic block because ATP contributes to the carbohydrate phosphorylation in the upper glycolysis and drives the synthesis of fructose-1,6-bisphosphate. Uncouplers inhibit the second pathway and, therefore, may increase the assimilation of Pi via the first route.

As experiments on animals are technically complicated, we tried to design a yeast-based system for identifying chemicals that decrease ATP levels across the widest possible range of non-toxic concentrations. We took advantage of a *Saccharomyces cerevisiae tps1* deletion mutant. *TPS1* is a gene coding for trehalose phosphate synthase, a protein acting to remove excessive intracellular glucose. Tps1p catalyzes the formation of trehalose phosphate, which inhibits hexokinase. In these ways, Tps1p regulates the rate of the upper glycolysis pathway and prevents glycolysis overflow [5]. Accordingly, glucose was shown to be toxic for the *Δtps1* strain [6,7]. The main reason why glucose addition to *Δtps1* cells blocks glycolysis is the following. Glucose conversion to glucose-6-phosphate and then to fructose-1,6-bisphosphate depletes the cellular stores of inorganic phosphate, thus inhibiting the formation of bisphosphoglycerate. It has been shown that upon the addition of glucose to *Δtps1* cells, the cellular ATP stock is significantly depleted: ATP is used to phosphorylate the sugars [8]. Nevertheless, based on the findings presented in [8], we reasoned that the stimulation of ATP hydrolysis or the prevention of its biosynthesis could alleviate the block. Indeed, if ATP molecules are used to phosphorylate either of the sugars, it strengthens the block. Moreover, the accumulation of sugar phosphates in the cytoplasm is toxic for the cells [9]. Besides, an excess of fructose-1,6-bisphosphate (FBP) hyperactivates Ras signaling, which eventually leads to cell death [10]. In the case that the ATP molecule is hydrolyzed (or not synthesized), it alleviates the block by (i) not supporting the phosphorylation and (ii) providing a substrate for the conversion of fructose-1,6-bisphosphate into bisphosphoglycerate (Figure 1). Consistent with this, it has been shown that cytoplasmic Pi is a limiting factor for the growth of tps1-delta cells in the presence of glucose [11,12] and that the inhibition of mitochondrial respiration rescues the glycolytic block [13].

We tried to adjust our experimental system to achieve only partial, not complete, ATP depletion, thus allowing cell growth. For that purpose, we used a growth medium containing a mixture of glucose at low concentration and a non-fermentable carbon source. We reasoned that, on the one hand, under such conditions, a decrease in the ATP level would first alleviate the block of glycolysis, thus allowing glucose depletion. On the other hand, in the case of mitochondrial ATP production still being possible, after glucose depletion, the cells would continue to grow using mitochondrial ATP synthesis fueled by the non-fermentable carbon source. Our current work points that, indeed, in such a system, a partial cellular de-energization by mild membrane depolarization stimulates cell growth and thus can be used for primary screening for the uncouplers of respiration and oxidative phosphorylation. We have also used our system for the initial characterization of two novel uncouplers, diethyl (tetradecyl) phenyl ammonium bromide (Kor105) and dodecyl diphenylamine (FS1).

## 2. Materials and Methods

### 2.1. Synthesis of FS1 (Dodecylmethyl Diphenylammonium)

A mixture of diphenylamine (3.313 g, 19.6 mM), 1-bromododecane (14.6 g, 58.6 mM) and KOH (6.86 g, 0.123 mM) in dimethylformamide (DMF) (50 mL) was stirred for 7 days at room temperature. The reaction mixture was diluted with dichloromethane (100 mL) and then washed with water four times, dried over Na_2_SO_4_ and evaporated *in vacuo*. The substance was dissolved in hexane (70 mL) and kept at 4 °C. Then, the precipitate was decanted by vacuum filtration and the filtrate was concentrated *in vacuo*. The procedure was repeated three times until the initial amine ceased to be determined by thin-layer chromatography (TLC) (hexane/ethyl acetate, 10:1). Hexane solution was passed through a thin layer of SiO_2_ and evaporated in vacuo to yield 3.5 g (53%) of dodecyldiphenylamine as an orange oil. 

The mixture of the obtained dodecyldiphenylamine (1.0 g, 3 mM) and methyl triflate (0.74 g, 4.5 mM) in benzene (2 mL) was heated at 75 °C for ten days. Then, methyl triflate was added again (0.250 g, 1.5 mmol) and the heating was continued for 12 days. The reaction mixture was cooled down to room temperature and washed with water three times. The aqueous phase was extracted with dichloromethane (3 × 5 mL). The combined organic phases were dried over Na_2_SO_4_ and evaporated in vacuo. The substance was dissolved in a minimal volume of dichloromethane, and then, an excess of hexane was added. The top phase was decanted; the oil was dissolved again in dichloromethane and treated with hexane to complete the precipitation. This procedure was repeated three times. The liquid was evaporated in vacuo. The final purification was conducted on a Reveleris^®^ X2-UV, using a dichloromethane–ethanol mixture (100:0→50:50) as the eluent. A 190 mg amount of the target compound (13%) was obtained after the evaporation of the solvents in vacuo. The LC-MS-calculated *m*/*z* for C25H38N+ is 352.30; an *m*/*z* of 352.06 was detected.

***Reagents used in the screening***. C4R1 – butyl [9-(2-ethoxycarbonylphenyl)- 6-(ethylamino)-2,7-dimethylxanthen-3-ylidene]-ethylazanium, C10R1 - decyl[9 -(2-ethoxycarbonylphenyl)-6-(ethylamino)-2,7-dimethylxanthen-3-ylidene]- ethylazaium, C12R1 dodecyl[9-(2-ethoxycarbonylphenyl)-6-(ethylamino)- 2,7-dimethylxanthen-3-ylidene]-ethylazanium, C4TPP butyltriphenylphosphonium, C8TPP - octyltriphenylphosphonium, C10TPP - decyltriphenylphosphonium, C_12_TPP - dodecyltriphenylphosphonium, C8Ber - octyl5,6-dihydro-9,10-dimethoxybenzo[g] -1,3-benzodioxolo[5–6-a]quinolizinium, SkQPal - 2,3-dimethyl-p-benzoquinone-dodecyl- 2,3,9,10-tetramethoxy-5,6-dihydroisoquinolino[2,1-b]isoquinolin-7-ium, SkQ1 - 2,3-dimethyl-p-benzoquinone- dodecyltriphenylphosphonium, and KOR105 -diethyl (tetradecyl) phenyl ammonium bromide were provided by G.A. Korshunova. FS1 — dodecylmethyl diphenylammonium—was synthesized by R.S. Kirsanov. BAC—benzalkonium chloride; DNP—2,4-dinitrophenol; PCP—pentachlorophenol; CCCP—carbonyl cyanide m-chlorophenyl hydrazone; FCCP—carbonyl cyanide-4-(trifluoromethoxy)phenylhydrazone; and NAC—N acetylcysteine—were obtained from Sigma.

***Yeast strains and growth analysis***. We used the W303-1A *Saccharomyces cerevisiae* yeast strain and its derivatives. Delta *tps1* was produced by the transformation of the *kanMX6:*:*Δtps1* gene cassette obtained by PCR from *Δtps1* from the Euroscarf collection. Primers: TPS1-F 5′-ACTGCACTGAGGTTCTAAGA, TPS1-R 5′-CGGGAGAGAAAGAAAGAGAG. The delta *tps1* strain was confirmed by PCR with an independent set of primers. The *Δlam1Δlam2Δlam3Δlam4* strain was described in [14]. To analyze the minimal inhibitory concentrations (MICs) of the chemicals for the wild-type and *Δlam1Δlam2Δlam3Δlam4* strains, cells taken from a logarithmically growing culture were adjusted to an optical density (OD, λ = 550 nm) of 0.05 (SpectrostarNano) and transferred as cell suspensions to a 96-well plate (Eppendorf). The cells were grown for 16 h at 30 °C and 750 rpm in a plate spectrophotometer with the following settings: temperature, 30 °C; plate shaking, 500 rpm. The OD at λ = 550 nm was measured using a SpectrostarNano. To compare the relative growth rates, we analyzed the increase in OD for the wild-type and the mutant strains.

***Glucose uptake measurement***. Yeast *Δtps1* cells taken from a logarithmic culture were adjusted to an OD of 1.0 (λ = 550 nm) in a SpectrostarNano. Glucose was added to a final concentration of 0.2%. The cell suspension was divided into 2 mL tubes, with 1 mL in each. Test substances were added to the medium. Ethanol, to a final concentration of 2%, was added as a solvent or separately, as a control. The glucose concentration in the medium was measured with a glucometer (OneTouch SelectSimple) after 30 min and after an hour. The glucose uptake rate was calculated as the decrease in glucose concentration induced by one OD unit of *Δtps1* cells in 1 mL of the medium per hour. In the absence of the additions, a *Δtps1* cells OD of 1.0 (λ = 550 nm) reduced the glucose concentration in the medium at a ~0.65 mM per hour rate.

***Analysis of the effects of the chemicals on tps1 growth in the presence of glucose***. Yeast *Δtps1* cells taken from a logarithmic culture grown on YPEtOH (YP-ethanol) were adjusted to an OD of 0.2 (λ = 550 nm) in a SpectrostarNano. Glucose was added to a 0.06% final concentration. Cell suspension was transferred to each well of a 48-well plate (Greiner), with 0.4 mL per well. Test substances were added to the medium. Ethanol was added to a final concentration of 2% as a solvent or separately. The cells were grown for 16 h at a temperature of 30 °C with plate shaking at 500 rpm on a SpectrostarNano device; the OD was measured (OD, λ = 550 nm) every 5 min.

***Measurements of yeast cell respiration***. Yeast cells were grown in liquid YPGly (YP-glycerol) medium overnight to OD540 = 0.5–1, pelleted for 5 min at 1125 g 4 °C, washed 2 times in chilled distilled water, resuspended in double-distilled water (1 g of wet weight per 300 μL) and stored on ice. Cell respiration was recorded using a Clarke-type electrode on a polarograph (Strathkelvin Instruments 782, United Kingdom) at 25 °C. The incubation medium contained 50 mM KH_2_PO_4_, pH 5.5, and 0.05% glucose. The OD of the cells in the polarographic cell was adjusted to OD 1–1.5. The ratio of the cell respiration rate in the presence of the chemicals to that in the absence (V/V0) was calculated.

***Isolation of rat liver mitochondria: respirometry***. Rat liver mitochondria were isolated by differential centrifugation [15] in a medium containing 250 mM sucrose, 5 mM MOPS (3-(N-morpholino)propanesulfonic acid, 4-morpholinepropanesulfonic acid), 1 mM EGTA (ethylene glycol-bis(β-aminoethyl ether)-N,N,N′,N′-tetraacetic acid), egtazic acid and 0.5 mg/mL bovine serum albumin, pH 7.4. The final wash was performed with the same medium. The protein concentration was determined by the Biuret method [16]. The handling of animals and experimental procedures were conducted in accordance with international guidelines for animal care and use and were approved by the Institutional Ethics Committee of the A.N. Belozersky Institute of Physico-Chemical Biology at Moscow State University (protocol #3 on February 12, 2018).

The respiration of the isolated mitochondria was measured using a standard polarographic technique with a Clark-type oxygen electrode (Strathkelvin Instruments, UK) at 25 °C using the 782 System software. The incubation medium contained 250 mM sucrose, 5 mM MOPS and 1 mM EGTA, pH 7.4. Succinate at a 5 mM concentration was used as a substrate. Every sample contained rotenone (2 μM) and oligomycin (1 μg/mL). The concentration of total mitochondrial protein was 0.8 mg/mL. Palmitic acid (1 μM) was added to FS1-containing samples.

### 2.2. Isolation of Yeast Mitochondria: Respirometry

We isolated mitochondria from the control (W303-1A) strains using the protocol described in [17]. Briefly, we first acquired yeast protoplasts by digesting the cells preincubated with dithiothreitol (DTT, 10 mg/g of yeast dry weight) with lyticase (2.5 mg/g of yeast wet weight). We then transferred the protoplasts to a hypotonic medium (mannitol, 0.3 M; EDTA, 1 mM; Tris−HCl, 1 mM; BSA, 4 mg/mL, pH = 7.2) to remove the plasma membranes. We isolated the mitochondrial fraction by differential centrifugation. We then measured the protein concentration with a Pierce™ BCA Protein Assay Kit (Thermo Fisher, cat. Number 23225) according to the manufacturer’s instructions.

The respiration of the isolated mitochondria was measured using a standard polarography technique with a Clark-type oxygen electrode (Strathkelvin Instruments 782, United Kingdom) at 25 °C using the DATLAB software. The incubation medium for the mitochondria contained 0.6 M mannitol, 10 mM Tris-HCl, 2 mM potassium acid (pH 7.4) and 15 mM pyruvate–malate (4:1).

## 3. Results

**Figure 2:**
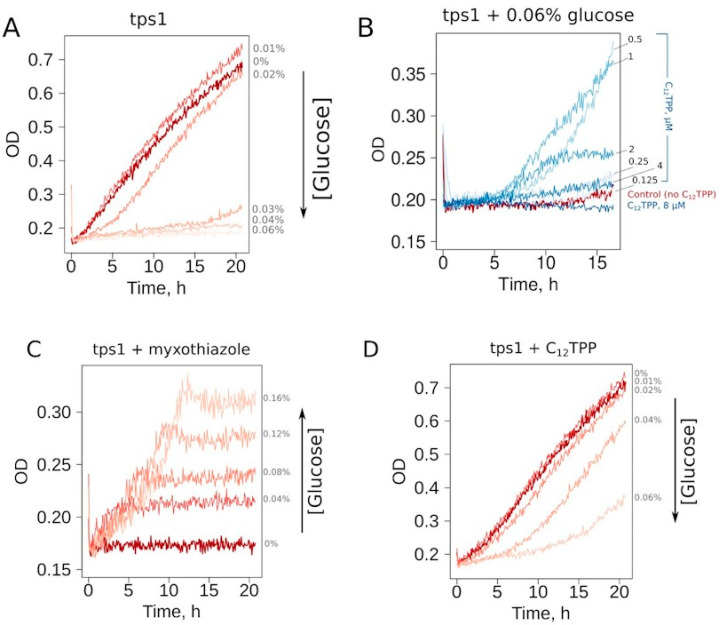
C_12_TPP and myxothiazol alleviate the glucose-induced inhibition of the growth of *tps1-delta* cells in a different fashion. The representative growth curves. (**A**) Titration of glucose. (**B**) Titration of C_12_TPP. (**C**) Titration of glucose in the presence of myxothiazol, 1 µM. (**D**) Titration of glucose in the presence of C_12_TPP, 1 µM.

First, we selected the growth conditions for *Δtps1* cells allowing the screening for the substances inducing a mild decrease in the ATP/ADP ratio. At the cell density of 10^6^ cell/mL, the addition of glucose to a 0.03% or higher concentration prevented the growth in ethanol-containing media (Figure 2A). To make our assay conditions sufficiently robust, we used a glucose concentration two times higher than that of the threshold level. Thus, the cells inoculated in the medium containing 0.06% glucose, 2% ethanol or 2% glycerol consistently did not show growth during overnight incubation at 30 °C. Since the mutant cells cannot be kept on solid YP-glycerol medium (we found that they stop growing after 2–3 passages), we used YP-ethanol media. In addition, most of the tested chemicals were dissolved in ethanol; the use of YP-ethanol media allowed us not to introduce an additional carbon source.

Representative curves show that the addition of a mild uncoupler C_12_TPP (dodecyltriphenylphosphonium) and the respiration inhibitor myxothiazol both allowed cell growth under these conditions (Figure 2B–D). Notably, the shapes of the growth curves were different: steep followed by flat in the case of myxothiazol (Figure 2C) and the opposite in the case of C_12_TPP (Figure 2D). Moreover, an increase in glucose concentration prevented C_12_TPP-induced growth but, on the contrary, stimulated that caused by myxothiazol addition (Figure 2C,D). Importantly, in the case of 0.04% glucose-containing media, C_12_TPP addition led to a higher final cell density than the addition of myxothiazol (Figure S1).

**Figure 3:**
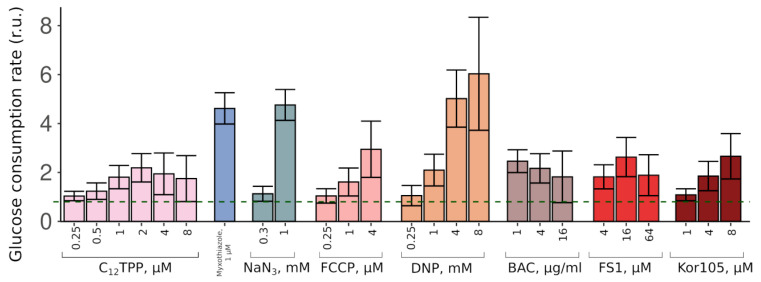
Glucose consumption rates of tps1-delta cells treated with various chemicals (normalized to the control). Error bars correspond to standard deviations; *n* = 3–8.

Thus, the two substances stimulated the growth in completely different fashions. These data suggest that while a complete inhibition of mitochondrial ATP production (myxothiazol) allows only relatively fast glucose-driven growth, partial inhibition causes a slow growth phase, which, upon sufficient glucose depletion, is followed by a fast glycerol-dependent growth. To test this, we measured the initial (during the first hour of incubation with the cells) rates of glucose consumption induced by C_12_TPP and myxothiazol. As expected, both substances stimulated glucose consumption. Consistent with the data on the levels of the initial growth stimulation, myxothiazol addition caused a significantly higher (approximately 5-fold) acceleration of glucose depletion from the medium than C_12_TPP (approximately 2-fold) (Figure 3).

**Figure 4:**
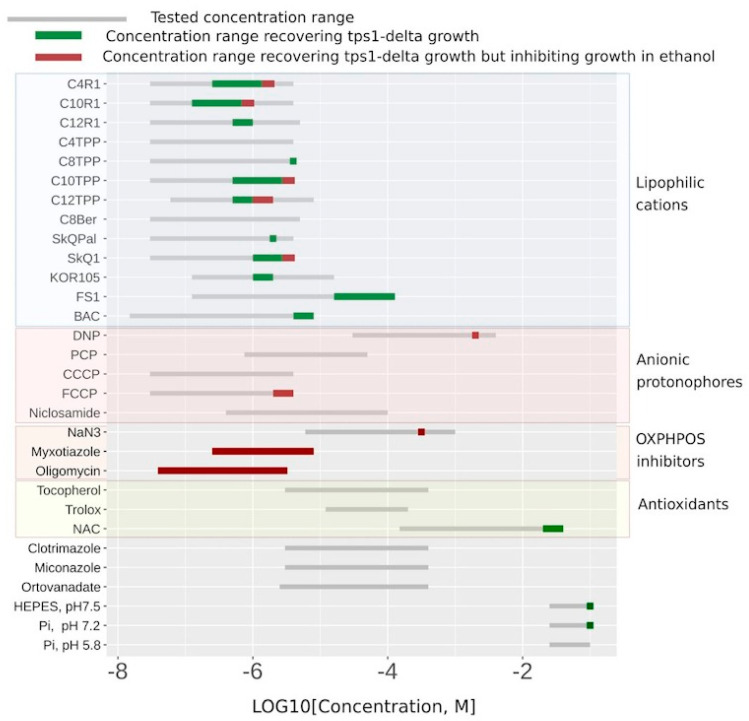
A list of chemicals tested for their ability to stimulate the growth of *tps1-delta* cells in the presence of glucose. The concentrations that did not produce any stimulation are shown in gray. The concentrations that correspond to the flat-followed-by-steep-shaped growth curves are shown in green; steep-followed-by-flat-shaped, in red.

As a proof of principle, we tested a number of uncouplers as well as several other compounds in our system. Figure 4 shows the substances that did display C_12_TPP-like behavior (flat-followed-by-steep growth curve) in green, the myxothiazol-like ones (steep-followed-by-flat growth curves) in red, and the ones that did not rescue at all in grey. While the ranges of the indicated concentrations for the substances shown in green correspond to flat-followed-by-steep-shaped curves of stimulated cell growth, the addition of higher concentrations of such substances resulted in steep-followed-by-flat growth curves (Figure 2B and Figure 4 and Table S1).

**Figure 5:**
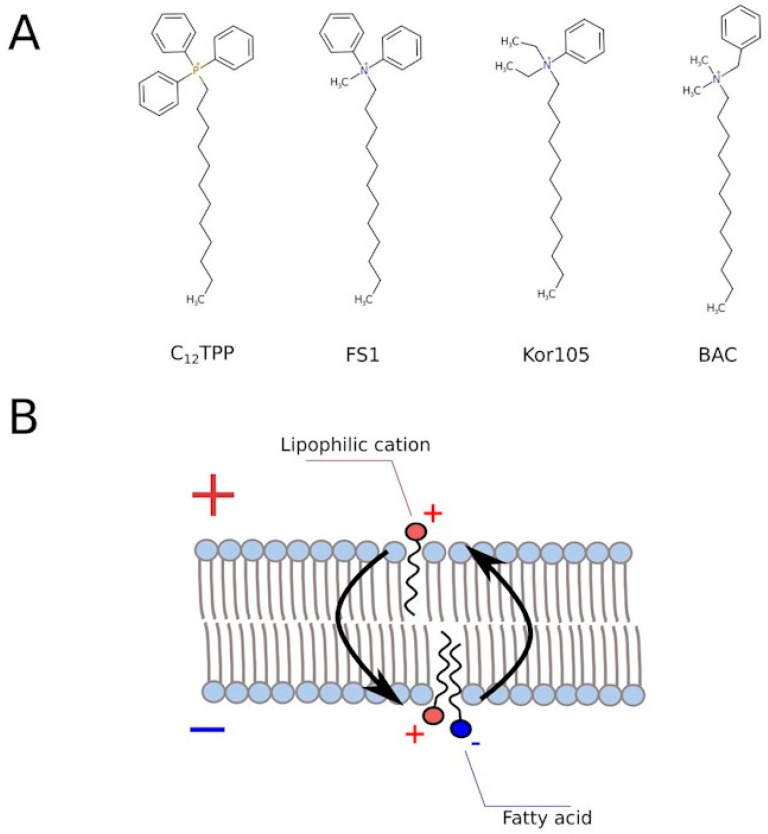
(**A**) Structural formulas of C_12_TPP and its analogs with varying degrees of charge shielding by phenyl groups. (**B**) A scheme illustrating the dissipation of the membrane potential by the lipophilic cations. “+” and “−” correspond to positive and negative electic charge, respectively.

Figure 4 shows that the lipophilic cations were generally more efficient in rescuing the growth than the conventional uncouplers (the uncouplers of both types are indicated in the table). Unlike the lipophilic cations, the conventional uncouplers appeared to possess myxothiazol-like effects in our assay (Figure S2), suggesting that the acting concentrations alleviated the glycolytic block but prevented the growth on the non-fermentable carbon source. Consistent with that, both FCCP (carbonyl cyanide-4-(trifluoromethoxy)phenylhydrazone) and DNP (2,4-dinitrophenol) stimulated the rate of glucose consumption (Figure 3). Importantly, NaN_3_, an inhibitor of respiration that is chemically different from myxothiazol, also stimulated both the steep-followed-by-flat-shaped growth (Figure 4) and the depletion of glucose (Figure 3). Based on this, we tested two novel compounds, potential mild uncouplers, Kor105 and FS1. The molecular formulas are shown in Figure 5A. These compounds are structurally similar to C_12_TPP, and the motivation for testing these compounds came from the molecular mechanism of C_12_TPP-mediated uncoupling. As we have shown earlier [18], the cycle of uncoupling includes the passage of a pair uncoupler-free fatty acid (FFA) across the membrane (Figure 5B). Apparently, as the positively charged phosphorus moiety of the C_12_TPP ion is shielded by the phenyl groups, the anion group of the FFA cannot come into close proximity with the cation moiety. This suggests that the passage of the pair is the rate-limiting step of the uncoupling. Indeed, being neutralized only partly, the anion group is likely to resist penetration through the membrane. In other words, it is possible that a C_12_TPP analog lacking one or two phenyl groups could be a more efficient uncoupler than C_12_TPP itself. Presently, we are testing these predictions using molecular dynamics simulations. To address this issue experimentally, we synthesized the corresponding molecules, and keeping in mind that phosphor-organic compounds are generally not recommended for the use on humans, the novel molecules contain nitrogen instead of phosphorus (Figure 5A). While the compound with one phenyl group, Kor105, has recently been described by us as a potential surfactant [19], FS1 is a novel substance. As shown by Figure 4, both compounds stimulated cell growth, with FS1 having a wider range of acting concentrations than Kor105 or C_12_TPP.

As expected, both FS1 and Kor105 stimulated glucose consumption; the effects were similar to those of C_12_TPP (Figure 3). Notably, when used at sufficiently high concentrations, some of the lipophilic cations induce steep-followed-by-flat (myxothiazol-like) growth curves (Figure 4). Possibly, in such cases, they act as general poisons. This means that they inhibit ATP production, in this way stimulating glucose consumption and, due to the poisoning, inhibiting the growth.

Next, we looked into the limitations of our screening system. Apparently, mild uncoupling is not the only way of alleviating the glycolytic block. Indeed, in agreement with the model of van Heerden and co-authors [8], the addition of alkaline phosphate buffer increased the initial rate of glucose consumption and boosted overnight cell growth (Figure 4). Moreover, according to [10], the alleviation of the glycolytic block is not the only means of protection against the toxicity of glucose. The authors showed that glucose addition causes hyper-activation of Ras in *∆tps1* cells, which eventually leads to the accumulation of reactive oxygen species (ROS) and cell death [10]. Consistent with their data, the addition of the antioxidant N acetylcysteine (NAC) allowed growth under our experimental conditions (Figure 4). Independently, it was shown that the loss of Tps1 activity resulted in a futile cycle of fructose-6-phosphate phosphorylation–dephosphorylation being activated, leading to ATP depletion [20]. Therefore, not only the substances decreasing the ATP/ADP ratio can stimulate the cell growth in our system, which points to the necessity of additional testing.

**Figure 6:**
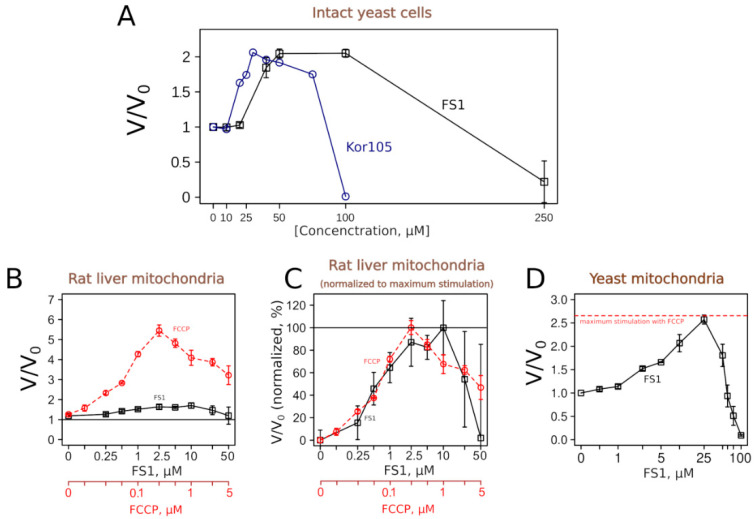
FS1 and Kor105 possess uncoupling activity. Respiration rates of intact yeast cells (**A**) and isolated yeast mitochondria (**D**) in the presence of different concentrations of the compounds, expressed as V divided by V0 (V, the rate in the presence of a compound, divided by V0, the rate without addition). (**B**,**C**) Stimulation of the respiration of rat liver mitochondria by FS1 and FCCP, absolute values of V divided by V0 and normalized ones (the maximal stimulation by a compound corresponds to 100%; the value without addition, i.e., without stimulation, corresponds to 0%), respectively. Error bars correspond to standard deviations; *n* = 3.

To test whether the analogs of C_12_TPP, Kor105 and FS1 uncouple respiration and phosphorylation, we measured their effects on respiration rates. Figure 6 shows that both substances, as expected, did induce an increase in the respiration rates of yeast cells. The concentration dependencies of the respiration rate stimulation were similar to the dependency for C_12_TPP [18].

To learn more about the uncoupling properties of FS1 and Kor105, we tested how they stimulated the respiration rates of mitochondria isolated from rat liver. It appeared that the respiration rate stimulation curves caused by the addition of the well-studied uncoupler C_12_TPP or Kor105 were rather similar, showing peaks of approximately 2-fold stimulation (Figure S3). On the contrary, FS1 addition produced a broad peak of approximately 10–50% stimulation (Figure 6). While the conventional uncoupler, FCCP, showed approximately the same width of the acting concentrations, the degree of the stimulation was much higher, approximately 6-fold (Figure 6). Importantly, it is believed that a small decrease in the membrane potential can cause a significant drop in mitochondrial ROS generation without affecting ATP synthesis (reviewed in [21,22,23]).

Surprisingly, our data showed that FS1 is more efficient in stimulating the respiration of intact yeast than when added to isolated mitochondria. As the purified mitochondria were obtained from the liver, we checked whether this was due to the differences between yeast and rat mitochondria. Thus, we tested the effect of FS1 on mitochondria purified from yeast. Figure 6D shows that FS1 was as efficient as FCCP in the stimulation of the respiration of isolated yeast mitochondria. As yeast and mammalian mitochondria differ in many ways, the reason for the relative inefficiency of the respiration rate stimulation of rat liver mitochondria by FS1 is a subject for an independent study.

Interestingly, benzalkonium chloride (BAC), a substance chemically similar to the lipophilic cations, also demonstrated a rescuing effect in our assay (Figure 4). We found the ranges of the acting concentrations of C_12_TPP and BAC to be approximately the same (Figure 4). Importantly, BAC did not stimulate respiration when added to mitochondria (Figure S3). Possibly, the rescuing effect of BAC was via mitochondrial ATP synthesis inhibition due to its interference with mitochondrial respiratory complexes. Indeed, recently, it has been shown that BAC inhibits respiration and ATP production when added to intact mitochondria [24]. In agreement with this, we found that BAC induced an approximately 3-fold stimulation of the rate of glucose consumption (Figure 3).

**Figure 7:**
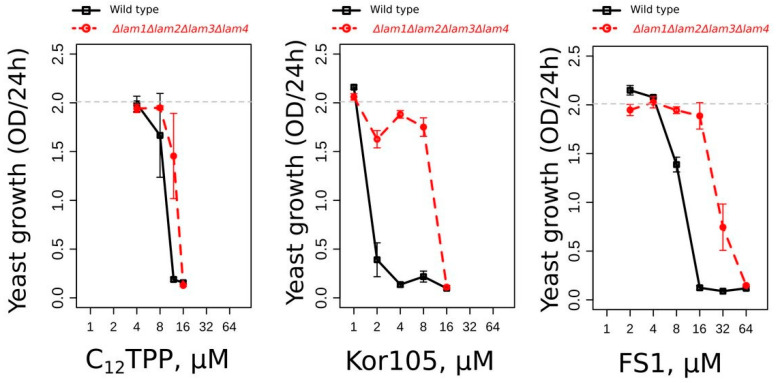
An increase in the plasma membrane (PM) ergosterol protects cells against the lipophilic cations. The graphs show the values of the cell densities grown for 24 h in the presence of the indicated concentrations of the cations. The wild-type and *lam1-4-delta* (the strain with elevated PM ergosterol) cells are compared on each graph. The cells were grown on YPD (YP-Dextrose) medium. Error bars correspond to standard deviations; *n* = 3.

Alternatively, it is possible that the rescuing effect of BAC, as well as the rescuing effects of C_12_TPP, FS1 and Kor105, was due to its action on the plasma membrane (PM). Recently, we have shown that mutants with elevated levels of plasma membrane ergosterol are more resistant to Kor105 and C_12_TPP than the wild type [19]. Similar to that of the other two substances, the toxicity of FS1 was also found to be lower in the mutant with elevated PM ergosterol (Figure 7). Our data indicate that the cations disturb the ordering of the plasma membrane lipid packaging, and this disturbance is dampened by ergosterol [19]. Possibly, ATP is consumed to repair the PM disturbance caused by the lipophilic cations.

## 4. Discussion

In our work, we present a novel screening system for non-toxic chemicals that interfere with cellular energy generation. As shown in Figure 4, the substances with membrane-depolarizing activity appear to be the most efficient in our system. Despite an obvious potential for the treatment of obesity and for the improvement of general metabolic health, currently, there are only two compounds with such activity that are approved for medical use. These compounds act primarily on mitochondrial inner membranes and thus can be defined as uncouplers of respiration and oxidative phosphorylation. One of them, niclosamide, is a common antihelminthic drug that is being repurposed for treating various other diseases (reviewed in [25,26]). Another one, sorafenib, is used to treat hepatic cancer [27,28]. Niclosamide was shown to work on mice kept on a high-fat diet to prevent obesity [29]. Pre-clinical studies also demonstrated that a recently described uncoupler, Bam15, is efficient in protecting mice against obesity and improving glycemic control [30].

The demand for medically relevant uncouplers is illustrated by the ongoing clinical trials of an old drug, DNP. This compound is probably the most well-studied mitochondrial uncoupler. It was shown that its usage as a weight loss agent in humans is accompanied by a set of negative side effects. Nevertheless, when used at lower, weight-neutral concentrations, it showed multiple positive effects in animal models of non-alcoholic steatohepatitis, diabetes and cardiovascular disease (reviewed in [31])**,** as well as in murine models of Parkinson disease (reviewed in [31])**.**

As mentioned above, our data indicate that lipophilic cations appear to be the most efficient in our system. Additionally, they show that the primary target of the cations is the PM (like in the case of BAC) and not mitochondria. This is surprising because such cations were shown to accumulate mainly in the mitochondria. Indeed, the uptake of lipophilic cations into mitochondria is driven by the mitochondrial membrane potential, leading to 100–500 fold accumulation in the matrix (reviewed in [32,33,34]). Possibly, upon treatment with the cations, the energetic cost of the PM repair is higher than the drop in mitochondrial ATP production.

Using this system, we have identified a novel uncoupling substance, FS1. One could notice an unusual feature of FS1′s action on respiration. While being rather efficient in stimulating the respiration of intact cells (more than two-fold, Figure 6A), it is relatively inefficient in stimulating the respiration of isolated rat liver mitochondria (approximately 1.5 fold, Figure 6B). In this respect, the conventional uncoupler, FCCP, is more efficient when used on the isolated rat liver mitochondria [35] than yeast cells [36]. Interestingly, FS1 was as efficient as FCCP when added to mitochondria isolated from yeast (Figure 6D), a fact pointing to some basic differences between yeast and mammalian mitochondria.

As discussed above, the rescuing activity of FS1 might be due to its disturbing action on the PM. More specifically, it is possible that the PM depolarization caused by FS1 stimulates the ATPase activity of the pumps maintaining the PM membrane potential. A significant amount of ATP might also be consumed to compensate for the PM depolarization caused by FS1.

At the same time, the data presented by Figure 7 indicate that the toxicity of FS1 is sensitive to the PM ergosterol. Indeed, ergosterol-rich domains may sequester FS1, as well as other lipocations, in the PM, thus preventing their shuttling across the membrane (the process required for depolarization). Notably, the depolarization of the yeast PM can induce a significant dissipation of sphingolipid-enriched, highly ordered microdomains within the membrane [37]. It was shown that these microdomains are essentially ergosterol-free [38], suggesting that the ergosterol-dependent sequestration of the lipophilic cations can preserve the integrity of the microdomains. Thus, it is possible to speculate that the interaction of the lipophilic cations with the PM strongly depends on the microdomain structure, sterol content and the initial value of the transmembrane potential. One cannot exclude that this also applies to conventional uncouplers. Indeed, one of the most commonly used uncouplers, FCCP, was shown to depolarize the PM [18,39,40] and to have several other effects apart from mitochondrial depolarization [41].

The rescuing effect of FS1 might also be explained by the chemical being a good substrate for MDR (multidrug resistance) pumps. Indeed, MDR activity is dependent on ATP hydrolysis [42], which may lead to the dissipation of the transmembrane potential due to the action of the ATP/ADP antiporter. Our observation that FS1 inhibits cell growth at much higher concentrations than Kor105 (Figure 4) is consistent with this idea. Notably, it is believed that the dissipation of the transmembrane potential via the stimulation of the mitochondrial ATP/ADP antiporter is the most physiological and thus the least damaging way of uncoupling ([43], also as a review). It is important to mention that the data indicating that the ATPase activity of yeast MDR transporters is not activated by the substrates [44] argue against this explanation. Still, one cannot exclude that the ATPase activity of a not-yet-identified pump responsible for FS1 extrusion is substrate-dependent.

Regardless of the particular reason for the rescuing effect, one can speculate that FS1 could be a lead substance for a novel type of medically relevant chemical compound inducing a mild decrease in the cellular ATP/ADP ratio.

## Supplementary Materials

The following are available online at https://www.mdpi.com/2218-273X/10/9/1345/s1. Figure S1: C_12_TPP addition led to a higher final cell density than the addition of myxothiazol in glucose-containing YPEtOH media, Figure S2: The effects of FCCP (A) and DNP (B) on the growth of *tps1-delta* cells in the glucose-containing YP ethanol medium, Figure S3: The effects of the lipophilic cations on the respiration rates of isolated mitochondria. Table S1: A list of chemicals tested for their ability to stimulate the growth of *tps1-delta* cells in the presence of glucose.
