# Supplementary material for: Lipophilic Cations Rescue the Growth of Yeast under the Conditions of Glycolysis Overflow
Source: Biomolecules. 2020 Sep 20;10(9):1345. doi: 10.3390/biom10091345 (PMC7563754; doi:10.3390/biom10091345)
Supplement: Supplementary file 1 [file biomolecules-10-01345-s001.zip › biomolecules-910400-Sup fig and tab/biomolecules-910400-Sup figures.pdf]

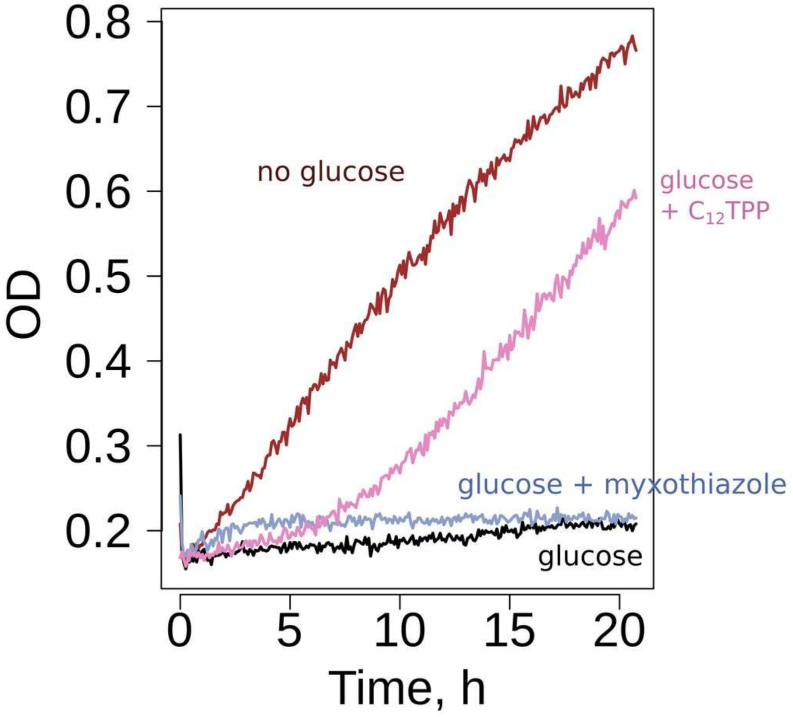

Figure S1. $C_{12}TPP$ addition led to a higher final cell density than the addition of myxothiazol in glucose-containing YPEtOH media.

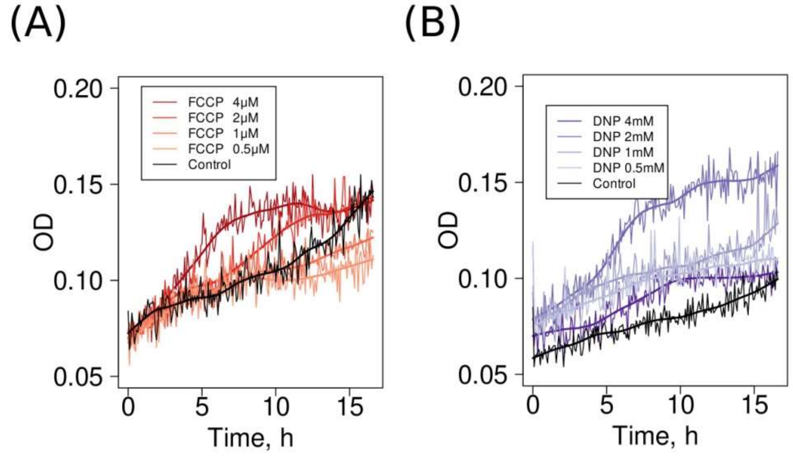

Figure S2. The effects of FCCP (A) and DNP (B) on the growth of *tps1-delta* cells in the glucose-containing YP ethanol medium.

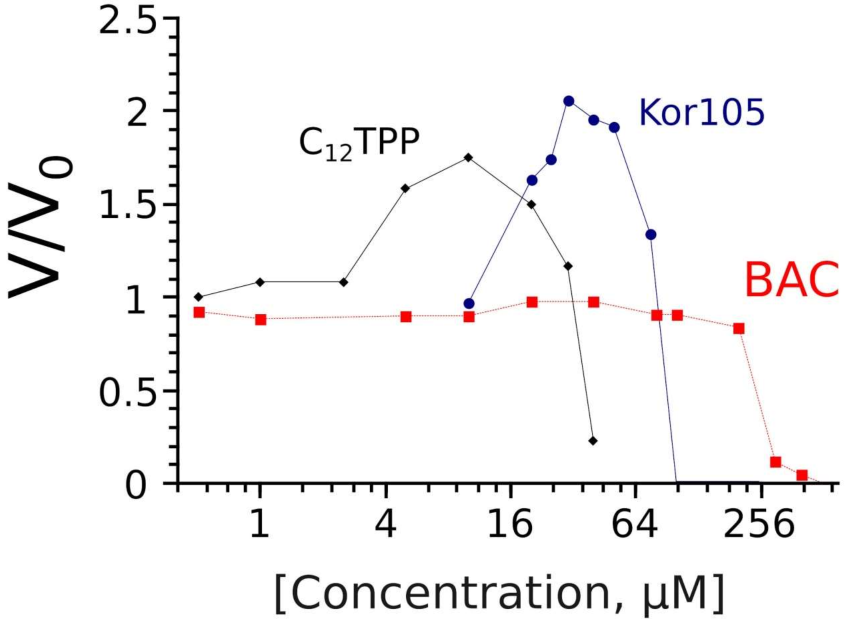

Figure S3. The effects of the lipophilic cations on the respiration rates of isolated mitochondria
